# The Combination of Whole Cell Lipidomics Analysis and Single Cell Confocal Imaging of Fluidity and Micropolarity Provides Insight into Stress-Induced Lipid Turnover in Subcellular Organelles of Pancreatic Beta Cells

**DOI:** 10.3390/molecules24203742

**Published:** 2019-10-17

**Authors:** Giuseppe Maulucci, Ofir Cohen, Bareket Daniel, Carla Ferreri, Shlomo Sasson

**Affiliations:** 1 Fondazione Policlinico Universitario A. Gemelli IRCSS, 00136 Rome, Italy; Giuseppe.Maulucci@unicatt.it; 2 Istituto di Fisica, Università Cattolica del Sacro Cuore, 00168 Rome, Italy; 3 Institute for Drug Research, Faculty of Medicine, The Hebrew University, 911210 Jerusalem, Israel; ofirco55@gmail.com (O.C.); bareketk@gmail.com (B.D.); 4 ISOF, Consiglio Nazionale delle Ricerche, 40129 Bologna, Italy; carla.ferreri@isof.cnr.it

**Keywords:** beta cells, diabetes, confocal microscopy, lipidomics, membrane fluidity maps, cell micropolarity maps

## Abstract

Modern omics techniques reveal molecular structures and cellular networks of tissues and cells in unprecedented detail. Recent advances in single cell analysis have further revolutionized all disciplines in cellular and molecular biology. These methods have also been employed in current investigations on the structure and function of insulin secreting beta cells under normal and pathological conditions that lead to an impaired glucose tolerance and type 2 diabetes. Proteomic and transcriptomic analyses have pointed to significant alterations in protein expression and function in beta cells exposed to diabetes like conditions (e.g., high glucose and/or saturated fatty acids levels). These nutritional overload stressful conditions are often defined as glucolipotoxic due to the progressive damage they cause to the cells. Our recent studies on the rat insulinoma-derived INS-1E beta cell line point to differential effects of such conditions in the phospholipid bilayers in beta cells. This review focuses on confocal microscopy-based detection of these profound alterations in the plasma membrane and membranes of insulin granules and lipid droplets in single beta cells under such nutritional load conditions.

## 1. Introduction

The composition of phospholipids in biological membranes determines their cell barrier and cellular communication functions as well as subcellular organelles structure and functions. These properties are determined by the nature of the various phospholipid species and the availability of free fatty acids (FFA) from cellular metabolism and the diet. Yet, the composition of phospholipids in membranes of different subcellular compartments in any given cell may differ greatly. For instance, MacDonald et al. [1] found significant changes in the distribution of phosphatidylserine (PS), phosphoinositol (PI), phosphatidylethanolamine (PE), phosphatidylcholine (PC), sphingomyelin (SM) amongst insulin granules, mitochondria and the whole insulin secreting beta cell (INS-1-832/13 cell line). Moreover, the abundance of different saturated (SFA), mono- (MUFA) and polyunsaturated fatty acids (PUFA) in these phospholipids also varied among the different compartments. Similarly interesting is the observation that glucose stimulation of beta cells induced reversible changes in the composition of fatty acid moieties of phospholipids in insulin granules. Such modifications and remodeled specific signatures of phospholipids in the membranes of insulin granules may alter their biophysical properties. This in turn may modify granules interactions with soluble proteins (e.g., SNAP receptors, SNAREs), with target membrane proteins within the granules (e.g., Vesicle associated membrane protein, VAMP) or with plasma membrane docking proteins (e.g., syntaxins) and affect insulin secretion. Indeed, Pearson et al. [2] observed changes in the turnover of arachidonic-containing phospholipids and diacylglycerols in glucose-stimulated beta cells. These findings have led to the theory that higher abundance of shorter length fatty acids and of unsaturated fatty acids in phospholipids may enhance the fusion and docking of insulin granules membrane bilayers to the plasma membrane upon glucose stimulation due to reversible changes in membrane fluidly and curvature. Moreover, glucolipotoxic conditions may also increase the oxidative burden and lead to endogenous oxidation of free and phospholipid-bound PUFA, as well as transforming *cis* configuration of double bonds to the *trans* configuration in their hydrocarbon backbone. This may lead to modified cellular functions, including insulin granule trafficking [3,4].

The basis for these theories was laid by earlier lipidomic investigations of beta cells, such as by Fex and Lernmark [5] or Cortizo et al. [6] who followed phospholipid turnover in resting and stimulated beta cells. Best et al. reviewed in 1984 [7] pioneering studies on the role of arachidonic acid metabolites in the regulation of beta cell function and insulin secretion. Metz suggested in 1986 [8] a key role for arachidonic acid metabolites in potentiating stimulus-secretion coupling in beta cells. Intensive research over the last 35 years have established significant roles of various enzymatic metabolites of arachidonic acid (e.g., prostaglandins, eicosanoids) and non-enzymatic products (e.g., 4-hydroxyalkenals) in the regulation of insulin secretion [9,10,11,12,13,14,15,16].

In addition to the inherent composition of phospholipids and their turnover in subcellular organelles in beta cells, it is equally important to emphasize the critical role of increased availability of dietary (essential and non-essential) FFA and their incorporation into phospholipids. This is of paramount consequence upon exposure of beta cells to high levels of SFA (e.g., palmitic acid) that ensues alone, or in combination with high glucose levels, an array of (gluco)lipotoxic effects that often contribute to the decline in the mass and function of beta cells in islets of Langerhans [17,18,19,20]

Our recent studies on the effect of high glucose and high palmitic acid levels on the phospholipid lipidome of rat insulinoma-derived INS-1E beta cells revealed profound changes in the abundance and distribution of various fatty acids in phospholipids. These studies reveal organelle-specific channeling of polyunsaturated fatty acids (PUFA), arachidonic acid in particular, to nonenzymatic peroxidation and the generation of 4hydroxyalkenals, which affect the cells in several ways [11,13]. Furthermore, advanced confocal microscopy imaging of the plasma membrane of the cells under such conditions detected minimal alterations in their biophysical properties. In contrast, membranes of insulin granules underwent significant remodeling that changed their fluidity. These methods also depicted neogenesis of lipid droplets in live cells upon exposure to excessive levels of palmitic acid [21,22,23]. This study aims at integrating these findings with standard lipidomics analyses to follow lipid turnover single beta cells and in their subcellular organelles and compartments.

## 2. Phospholipid Turnover in Cells

The fatty acid composition in membrane phospholipids is constantly remodeled by the influence of free fatty acid availability, enzymatic activity of phospholipases, stressful condition (e.g., nutritional deficiencies or overload conditions) or metabolic diseases. The remodeling is a dynamic and fast process that changes the equilibrium between fatty acid hydrolysis from phospholipids by phospholipase A2 (PLA_2_), on one hand, and their acylation to the phospholipid backbone by lysophospholipid acyl transferase (LPAT), on the other [24]. Once PUFA are hydrolyzed from the phospholipid backbone they serve as substrates for enzymatic conversions to plethora of metabolites. Hitherto, hundreds metabolites of arachidonic acid and other PUFA have been identified, many of which constitute distinct groups of ligands to known receptors and transcription factors [12,25,26,27,28]. Different mammalian cells express enzymatic pathways that convert arachidonic acid and other PUFA to discrete cell-specific repertoire of bioactive metabolites in a cell-specific manner. These metabolites subsequently regulate various cellular functions in autocrine and/or paracrine fashions. It has been shown that endogenous PUFA metabolites, such as 20-hydroperoxyeicosatetraenoic acid (20-HETE), prostaglandin E1, E3, J2 and I2, or endocannabinoids regulate beta cell functions [14,16,29,30,31,32,33,34,35,36,37,38,39]. Some of these mediators are also generated in beta cells by direct enzymatic transformation of exogenously available unsaturated fatty acids; it has been shown that certain metabolites improved insulin secretion and ameliorate obesity- and cytokine-induced beta cell damage [16,40,41]. Equally important are the findings that assigned key regulatory roles for activated fatty acid receptors, such as GPR41, in modulating insulin secretion upon binding of fatty acid ligands [42,43,44,45,46,47,48]. In addition, it has been shown that intracellular n-3 PUFA transformation by elongases (e.g., docosahexaenoic acid formation) may protect against glucolipotoxicity-induced apoptosis in rodent and human islets [49]. Nonetheless, Johnston et al. [50] have recently pointed to an association between long-term increase in circulating non-esterified fatty acids and lower beta cell function.

The enzymatic conversions of PUFA occur in cells along with non-enzymatic transformations. The potency of these non-enzymatic pathways is determined foremost by the levels of oxygen free radicals, which initiate the peroxidation of PUFA and lead to the generation of a group of chemically and biologically reactive aldehydes, of which 4-hydroxyalkenals are prominent. There are two contrasting effects of 4-hydroxyalkenals in cells: numerous studies have shown that these reactive electrophiles form adducts with macromolecules, alter their function and contribute to the etiology and progression of pathological processes [51]. However, when present at physiological and non-toxic levels they interact with receptors and ligand-activated transcription factors in a specific manner and modulate cell functions in autocrine or paracrine manners. Indeed, Poganik el al. [52] have recently compiled evidence to propose that native reactive electrophiles (e.g., 4-hydroxyalkenals) are signaling molecules. Moreover, some of these intracellular interactions were found to evoke hormetic responses that induced or augmented cellular defense mechanisms that ultimately enhance the elimination of the same electrophiles [12,23,51,53,54,55,56,57].

These studies were mostly based upon whole cell lipidomic analyses that usually do not detect subcellular membrane-specific phospholipid turnover. Apparently, disparate remodeling of phospholipids in subcellular compartments or organelles may affect cellular functions in various ways. Monitoring and understanding such variable subcellular remodeling may reveal for instance: (i) whether plasma membranes of cells are inherently protected against major remodeling of phospholipids and thus preserve their barrier and communication capabilities with the surrounding environment; (ii) to what extent the remodeling of mitochondria membranes may affect their permeability, membrane potential and oxidative phosphorylation capacity; (iii) how the remodeling of phospholipids in the ER modulates protein sorting and chaperoning or alters their capacity to generate mono-layered lipid droplets within this compartment; (iv) could lysosomal function be influenced upon phospholipid remodeling or (v) to what level neurotransmitter or hormone secretion from vesicles or granules in neurons and endocrine cells, respectively, is disrupted of enhanced due to alterations in their tethering, docking and fusion with the plasma membrane and subsequent internalization. Tedious fractionation, separation and isolation techniques of subcellular organelle fractions were practiced in the past to answer such questions. Often, the amount and cross-contamination of the isolated fractions resulted in erroneous analyses. We showed that what was considered a standard and efficient purification method of the plasma membrane fraction of skeletal muscle cells carried in fact a substantial cross-contamination of intracellular microsomal membrane that could greatly obscure the experimental results [58].

## 3. Lipidomic Analyses of Beta Cells

**Figure 1:**
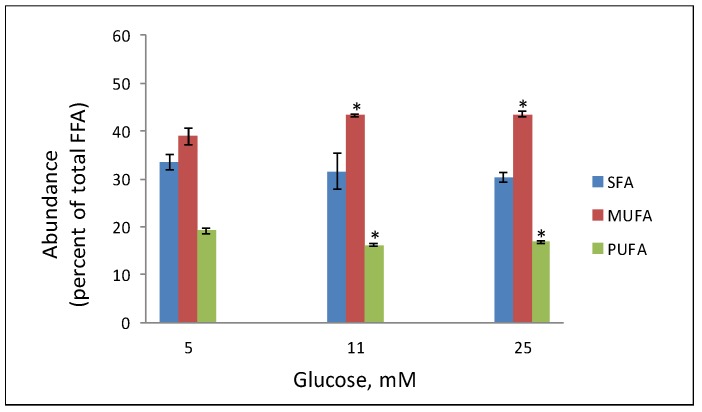
Glucose-induced remodeling of phospholipid in INS-1E cells. INS-1E cells were incubated in serum-free medium supplemented with the indicated glucose concentration for 16 h, and processed for lipidomics analysis as described [13]. The abundance of Saturated- (SFA), Monounsaturated- (MUFA) and polyunsaturated fatty acids (PUFA) is given as percent of total fatty acid content. Mean ± SEM, *n* = 4. * *p* < 0.05 significantly different from the corresponding abundance at the 5 mM glucose incubation (adapted from [13]).

We have employed non-targeted lipidomics analysis to study the impact of high glucose and high palmitic acid levels on the turnover of fatty acids in phospholipids of INS-1E cells. These studies discovered significant changes in the content of SFA, MUFA and PUFA [13]. Figure 1 shows the changes in their abundance after exposure of the cells to 11 and 25 mM glucose in comparison to cells that were maintained at 5 mM glucose. The abundance of PUFA was significantly decreased, MUFA increased and SFA levels remained constant under the high glucose incubations. Noteworthy, the total fatty acid content remained unaltered under these experimental conditions. This study also showed that that the released PUFA (i.e., arachidonic and linoleic acids) were avidly peroxidized to 4-hydroxynonenal (4-HNE). The latter in turn activated peroxisome proliferator-activated receptor-δ (PPARδ) that further augmented glucose-stimulated insulin secretion (GSIS). Thus, increasing glucose concentrations have not been previously considered to have specific stressful effects on membranes; in fact, 5 or 11 mM glucose were indifferently reported for beta cell culture conditions without affecting cell viability. In our study we showed for the first time that cellular membranes were not just spectators but were the source for lipid precursors of signaling molecules such as PUFA.

**Figure 2:**
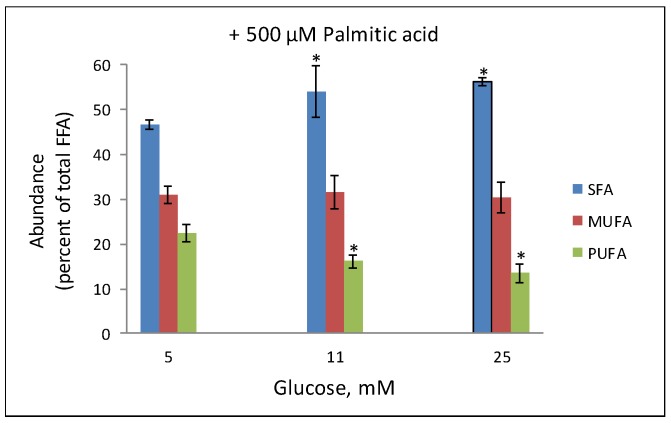
Impact of palmitic acid on fatty acid abundance in phospholipids of INS-1E cells. INS-1E cells were incubated in serum-free medium supplemented with the indicated glucose concentration for 32 h. Palmitic acid (500 µM) was added during the last 16 h of incubation. The cells were then harvested and processed for lipidomics analysis as described [11]. The abundance of SFA, MUFA and PUFA is given as percent of total fatty acid content. Mean ± SEM, *n* = 4. * *p* < 0.05 significantly different from the corresponding values at the 5 mM glucose incubation (adapted from [11]).

Concomitant exposure of the cells to increasing levels of palmitic acid and glucose further modified the abundance of fatty acids in phospholipids. Figure 2 shows the expected increase of the abundance of SFA (i.e., palmitic acid) that was accompanied with nearly 50% depletion in the amount of PUFA in phospholipids. Increasing glucose levels in the incubation intensified these palmitic acid-induced phospholipid remodeling effects. This study also showed that at these ranges the peroxidation of the released arachidonic and linoleic acids to 4-HNE also activated PPARδ and evoked augmented GSIS [11]. It is important to note that the upper limit of non-toxic concentrations of palmitic acid that did not compromise cell viability upon prolonged incubations were 300, 150, and 100 µM at 5, 11 and 25 mM glucose, respectively.

As mentioned above, these whole cell lipidomic analyses could not detect changes in fatty acid composition in membranes of subcellular compartments in the cells. This limitation also applies to other whole-cell analyses, such as shotgun lipidomics [59]. This method detects and reports the cellular content of most commonly known lipids in cells such as, free fatty acids and their metabolites (e.g., eicosanoids), glycerophospholipids (e.g., PC, PE, PS, PG, PI, PA), glycerolipids (e.g., TAG, DAG, MAG), diphosphatidylglycerol lipids (cardiolipins), sphingolipids (e.g., sphingomyelin, sphingosines, ceramides, cerebrosides, gangliosides) or sterol lipids (e.g., steroids, sterols) [60]. Several studies used this technique in diabetes research and found alterations in myocardial cardiolipin content and composition at the early stages of the disease [61]. Others correlated alterations in the plasma lipidome of diabetic patients or in tissues of diabetic mice (ob/ob) to the progression of impaired glucose tolerance [62,63]. Nevertheless, such shotgun lipidomic analyses have not yet revealed alterations in subcellular compartments of pancreatic beta cells under normal or stressful stimuli.

Other mass spectrometry methods have also been used to study beta cells. Recent temporal analysis of palmitic acid-treated INS-1 beta cells was based on isobaric labeling-based mass spectrometry and bioinformatics [64]. It highlighted altered cholesterol and fatty acid metabolism as early toxic events associated with ER stress. This quantitative strategy provided insight into general molecular events (lipid metabolism) and pathway adaptation in the cells. Other groups employed non-targeted mass-spectrometric lipidomics to study beta cells [65]. For instance, electrospray ionization mass spectrometric analysis that was employed to analyze phospholipids in INS-1 beta cells [66] discovered changes in total PUFA and MUFA contents, which were quite similar to the results of the lipidomics analysis we have performed, as described above. Interestingly, treatment of the cells with palmitic acid in this study had little effect on the content of both classes of fatty acids in the cells. Recent advances in mass spectrometric methods, such as the matrix-assisted laser desorption/ionization (MALDI) imaging mass spectroscopy (IMS), have been used to obtain molecular profiling of mouse pancreatic tissues [67]. Immunofluorescent images that were acquired from serial pancreatic sections were co-registered with the MS images of the sections and enabled molecular identification of specific phospholipid and glycolipid isoforms. The region selective molecular specificity afforded by this method revealed profound differences between endocrine and exocrine cells. Yet, the capacity of the method to detect clearly changes in the distribution of lipids within insulin granules or other subcellular organelles remains limited. Others have employed nanospray desorption electrospray ionization mass spectrometry imaging (nano-DESI-MSI) to identify different lipid classes in individual islets of Langerhans and the surrounding exocrine cells in sections of mouse pancreatic tissues [68]. The study found some disparate distribution of certain lipid species (including PUFA rich phospholipids, such as PC 34:2, PC 36:2, PC 36:4, PC 38:4) between the two types of cells. Using this method for the analysis of pancreatic tissues from normal and diabetic animals may enable estimation of the content of the phospholipids and other lipids. Both the MALDI-IMS and nano-DESI-MSI techniques analyze islets in pancreatic tissue sections without discriminating amongst the different types of endocrine cells (alpha, beta and delta cells) and without providing clear intracellular maps of the distribution of the different lipid species in subcellular organelles. Recent advances in enhancing the power of resolution of such single-cell analysis by different mass spectrometric platforms may contribute to comprehensive analysis of lipid turnover in beta cells [69].

## 4. Confocal Imaging-Based Fluidity and Micropolarity Maps of Single Beta Cells

Our interests in ascertaining the impact of phospholipid remodeling in the beta cell lipidome under nutritional overload conditions led to two independent confocal imaging strategies of INS-1E beta cells. The first employed spectral analysis of the fluorescent probe Laurdan to provide fluidity maps of single cell membranes [22]. The second exploited the micropolarity-sensitive emission profile of the dye Nile red to give intracellular maps of neutral and polar lipids in subcellular organelles [21].

**Figure 3:**
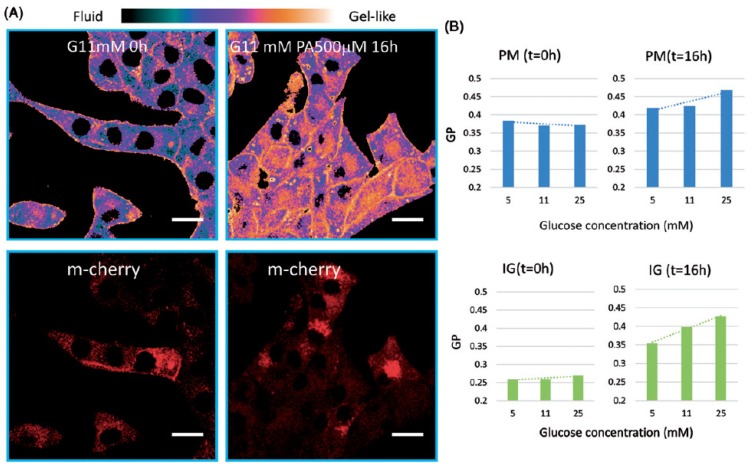
Generalized Polarization (GP) values of the plasma membranes (PM) and insulin granules (IG, mCherry positive organelles) in glucose treated INS-1E cells. (**A**), Representative high-resolution fluorescence images of Laurdan emission for fluidity investigation along with mCherry emission images in INS-1E cells exposed to 11 mM glucose for 32 h and 500 µM palmitic acid (PA) during the last 16 h of incubation. mCherry labeled insulin granules, which have spherical shapes of about 0.5–1 μm diameter. Scale bar is 10 μm. (**B**) Summary of GP values of plasma membrane and insulin granules in cells exposed to different glucose levels without (left) and with palmitic acid (right). Copied with permission from [22].

In the first case the advantages of fluorescence temporal imaging-based detection methods were exploited to obtain high resolution imaging of subcellular organelles in beta cells. For this purpose, we expressed in INS-1E cells IAPP-mCherry protein that is targeted to insulin granules [70] (probe courtesy of Dr. Patrick E. MacDonald, University of Alberta, Edmonton, Canada). mCherry-expressing cells were incubated at 5, 11 and 25 mM and loaded with the fluorescent dye Laurdan, which integrates into lipid phases in membranes. Its excited-state relaxation is highly sensitive to the presence and mobility of water molecules within the membrane bilayer, while being insensitive to the head-group type in phospholipids [71,72]. By using two-photon infrared excitation techniques and dual-wavelength ratio measurements, we detected Laurdan emission spectrum of coexisting lipid domains in the cells and obtained information on membrane fluidity by following the shift from ordered (gel) phases (yellow-orange emission) to disordered (liquid-crystalline) phases (violet-purple emission). Membrane fluidity in the confocal images was then reported in terms of ratio of emission intensities for each pixel by using Generalized Polarization (GP) value. This is defined as GP = (*I*_G_ − *I*_R_)/(*I*_G_ + *I*_R_); *I*_G_, emission in the range of 400–460 nm; *I*_R_, emission at the range of 470–530 nm. The GP value ranges from −1 (fluid, liquid disordered state) to 1 (gel-like, solid ordered state). Figure 3 shows such Laurdan spectral analysis of mCherry expressing INS-1E cells that were incubated at 5, 11 and 25 mm glucose for 32 h without or with 500 µM palmitic acid during the last 16 h of incubation. 

The fluidity maps of plasma membranes revealed that the GP values remained unaltered (GP = 0.38) in cells that were incubated with increasing glucose concentrations. This indicates that the release of PUFA from phospholipids in the cell sunder high glucose conditions (reported in [13]) did not involve significant remodeling of the plasma membrane. Thus, the barrier and communication properties of the plasma membrane, as well as the capacity to interact with secretory insulin granules upon glucose stimulation were preserved. Of interest are the lower GP values (0.26) of the insulin granules (mCherry positive organelles) that are indicative of a more fluid state than that of the plasma membranes, at all glucose concentrations. This seems to result from a higher abundance of PUFA and the corresponding liquid disordered phase. The latter results from the non-linear geometrical configuration of double bonds in *cis* positions [73]. The GP value increased slightly following the incubation with 11 and 25 mM glucose due to the hydrolysis of PUFA from phospholipids, as we observed in the abovementioned lipidomics analysis. The incubation with 500 µM palmitic acid had significant effects on the GP value in plasma membranes and insulin granules, which steadily increased in both compartments in a glucose-dependent manner. These dramatic changes in membrane fluidity of insulin granules reflect well the capacity of the incorporated saturated palmitic acid to induce transition to a solid-ordered gel-like phase of phospholipids in membrane bilayers. Furthermore, depletion of PUFA from insulin granules’ phospholipids may also contribute to the higher levels of the observed GP values. Indeed, this scenario correlates well with the results on the lipidomics analysis in similarly-treated cells that showed marked loss of PUFA from phospholipids (Figure 2). The impact of this phenomenon on the recruitment of insulin granules for secretion upon glucose stimulation is complex. We have recently shown [73] that non-toxic palmitic acid levels augmented GSIS, enabling the organism to facilitate insulin-mediated glucose and fatty acid disposal and thereby reduce the risk of developing peripheral diabetes complications. However, once reaching the toxic range of palmitic acid concentrations, which is reciprocally related to increasing glucose concentrations, the insulin secretory capacity is impaired, partly due to the rigidification of the insulin granule membranes. This study shows that Laurdan-based fluidity maps of cells complements with whole cell lipidomic analysis and provides insight to localized remodeling of phospholipids (i.e., plasma membranes and insulin granules).

**Figure 4:**
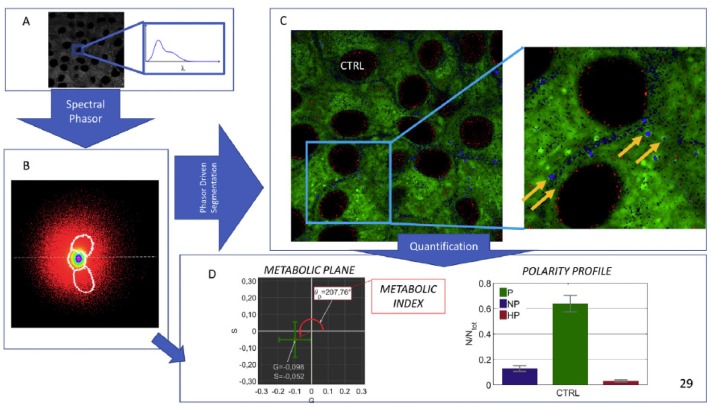
Workflow of the method based on the phasor driven segmentation of Nile Red spectral images. INS-1E cells were maintained at 11 mM glucose received 300 µM palmitic acid for 16 h. (**A**), Nile Red spectral images of live INS-1E cells. Each pixel of the spectral image is associated with the Nile Red emission spectrum. (**B**), Phasor plot generation: the cloud appears as broad and elliptical, because the co-existence of the three classes of lipids (NP, neutral lipids; P, polar lipids and HP, highly polar lipids), which are simultaneously present in the cells. By selecting on the phasor plane the domains corresponding to the three classes (white lines), it becomes possible to remap them to the original fluorescence image. (**C**), Segmentation of the three lipid classes: NP are reported in Blue, P in Green, HP in red. In the magnification, lipid droplets are visualized as spherical particles. P lipids are also localized in the plasma membrane, of which phospholipids is the main component. (**D**), Extraction of the angle θP formed by the center of mass of the cloud in the phasor plot with the g-axis provides information about the average polarity. From the segmented channels, the fractional contribution of the different lipid classes, in terms of the relative fraction of pixels belonging to a particular class was retrieved. Copied with permission from [21].

In the second case the solvatochromic and lipophilic properties of the fluorescent probe Nile Red were exploited to obtain high-resolution micropolarity maps of individual INS-1E cells [21]. The probe exhibits an emission shift from yellow to red when the degree of polarity of the lipid environment increases [74]. This property enables the detection of the degree of polarity of lipids in cells by evaluating the quantitative ratio of red and yellow emissions. In this study INS-1E cells were incubated with 1 μM of Nile Red for 30 min in the dark and then placed on the inverted confocal microscope equipped with a live chamber and 32 channel spectral images were obtained using a 60X objective under 488 nm excitation for the probe. Internal photon multiplier tubes collected images in 16-bit, unsigned images at 0.25 ms dwell time. This procedure, which enables the assessment of differences of the polarity of the various compartments in the cell, allows to refine the investigation of the subcellular distribution of neutral and polar lipids [75,76]. Figure 4 depicts the principles of phasor analysis that was employed to analyze images of Nile red-treated cells. The method is explained in details in our recent study [21] and elsewhere [77,78]. The cells that were incubated with 300 µM palmitic acid handled the increased influx of palmitic acid by incorporating it into triglycerides that were then sequestered in newly formed lipid droplets.

In the magnification (Figure 4) it is shown that the lipid droplets are composed of a core of non-polar (NP) lipids (blue spots; triglycerides) and a surrounding monolayer of polar (P) lipids (green coating), which is typical of lipid droplets [79]. This analysis also revealed that P lipids are localized in the plasma membrane. Interestingly, hyperpolar (HP) lipids are associated with nuclear membranes, or unevenly compartmentalized in internal membranes throughout the cytoplasm. We propose that these compartments may also serve a major target for phospholipid turnover in nutritionally-challenged beta cells and could therefore be the source for the PUFA required for non-enzymatic peroxidation and generation of 4-HNE. This method, which is very useful for lipid droplet research, can also be applied to Nile red spectral analysis of phospholipid remodeling in subcellular compartments. This may be used for instance for simultaneous fluorescent labeling of insulin granules and other organelles. While mCherry labeling may pose limitation due to overlapping emission spectrum with Nile red, other probes, such as phogrin-fluorescents proteins [80] or neuropeptide Y-pHluorin [81] may be useful for labelling the granules. 

## 5. Conclusions

Current lipidomic analyses of beta cells show that hyperglycemic- and hyperlipidemic-like conditions induce fast remodeling of phospholipids. These modifications may reflect substantial structural and functional changes in the cells. These methods have not yet progressed to allow for subcellular lipidomic analysis in fixed or lived cells. Confocal imaging that provide high resolution maps of subcellular membrane fluidity and lipid micropolarity maps of live cells may complement the lipidomic analyses by depicting membrane remodeling upon various stressful stimuli. Our recent results in lipidomics and lipid imaging in beta cells highlight this potential. This has also been demonstrated in a study that employed mass-spectrometry based oxidative lipidomics and lipid imaging in traumatic brain injury models [82]. Finally, recent reports on beta cells heterogeneity [83] attest to the limitations of the whole cell lipidomic analysis and the clear advantages of individual cell analysis by confocal imaging used in this study, which may distinguish among different beta cell populations in islets of Langerhans.
